# An Address

**Published:** 1864-02

**Authors:** H. Benedict


### DENTAL REGISTER OF THE WEST.

VOL. XVIII.] FEBRUARY, 1864. [No. 2.

### Original Essays and Communications.

### AN ADDRESS.

Read before the Mechigan State Dental Association, January, 1864.—BY DR. H. BENEDICT.

*Mr. President and Fellow Dentists:*—On retiring from this Chair, which by your kindness I have had the honor of filling for the last year, it is expected of me to present you with some kind of an Address. I must acknowledge my inability to do so in a manner I could wish. But, a few thoughts may not be amiss; and if they excite us to greater diligence and a closer application to our professional duties for the benefit of our fellow-beings, it will be a suitable reward.

Let me call your attention to the great difference there is in our profession now, and what it was thirty or forty years since. Then, dentistry was looked upon as a sort of trade or catch-penny calling, that any ignoramus could—with an old turn-key, a dozen or two of other instruments, and an old bone out of which to carve his teeth, all snugly packed in a tin box—tramp from house to house and pick up a few more dimes than he could by chopping cord-wood. And here, as a sample, let me show you the production of one of these customers, and, I might almost say, pupils of one of that class. These teeth were manufactured and worn several years by a Mr. B., then living in Rochester, N. Y., but now a resident of Detroit, Mich.

The circumstances were these: A dentist called on Mr. B., and wished to supply the place of the lost teeth in Mr. B.'s mouth, with a very nice set that he could make. Mr. B. consented; and, after some time spent in sawing, filing and boring, succeeded in fastening, by wires to the old roots and other teeth, what he called "a beautiful denture.,' Mr. B. paid and dismissed the dentist; but having watched the proceeding, and not being fully satisfied with the job, hunted up some old bones and commenced the practice of dentistry; and in about the same time that the veritable dentist took to accomplish his job, Mr. B. made and fitted this, which he says looked and felt much better than the other. It was replaced by a set on gold, a few years since, made by Dr. W., a former partner of mine. Such then was Mechanical Dentistry; and the Operative branch was, in most cases, on a par with it. But what a change, when we look at the beautiful and nicely-adapted teeth now made by our best dentists. And again, when we compare the medical, surgical and operative skill now displayed in protecting and redeeming the natural teeth and surrounding parts from disease and decay, and in replacing the lost portions of dentine and enamel with gold, so manipulated as to restore the tooth to its former shape and usefulness—even if the crown be nearly or quite gone—and in so treating the surrounding parts as to eradicate disease and cause nature to restore lost portions of the maxillary and other bones, so that the contour of the parts are restored, and teeth loosened by the loss of the alveola are again made firm and good, and the whole taking on a normal and healthy action. [See Dr. W. H. Atkinson's Essay on Dental Pathology and Surgery, and remarks on the same by the members of the American Dental Association; and for Operative Dentistry, see case reported by W. H. Allen to the same Association.]

But when we come to examine ourselves, and look upon others from our stand-point, we are surprised to see the indifference manifiested by a large portion of the dentists to the true merits and high standing of our profession. Are we not too conceited, and think we are about or quite equal to the best? If we see our own defects, and are striving to overcome them by study and associations with others who are on the same road either ahead or behind us, it gives us a more extended view of the lack of general interest there is in and to the true calling of our profession.

Out of about one hundred and fifty who style themselves dentists, and are practicing in this State, how many are here to-day? and how many even of the members who have united with us since this Association was formed, are present? Why is it? have they nothing to learn? or if they have, concluded that they can not learn it here?

If so, I for one would be glad to meet them here, and would willingly keep silent while they reflect from their brilliant intellects, that light we are all aiming for.

And this calls to mind a certain young dentist who graduated on a six-months' tuition in a dental office, and puts on considerable style, and is doing in his *way* a fair business. When learning of an operation performed by Dr. W. H. Atkinson of New York, and reported at the Saratoga Convention last summer, pronounced it all "humbug," and that "such operations never amount to anything." He is also entirely above attending an Association or Convention of dentists, as they amount to nothing more than a chance for a few self-conceited ones to gas, and make fee-bills which they would violate,—showing that he thought the only object we have in meeting here, is to talk over fees and fee-bills; that the whole had been learned that could be. How many such can you call to mind?

Yet, how gladly would we take them by the hand and work, shoulder to shoulder with them, for the advancement of our profession and their elevation, if they only could be convinced of the need of it and would come and help us. Which one of us have not been benefitted by our Michigan Dental Association? Who is there here that would vote to disband? Would it not be better for us, and for our patrons, if every true dentist in the State was an earnest and faithful member with us? If such was the case, we could form local Societies in several parts of the State; when we could meet oftener; and what would sooner separate the true and honest dentist who is working for the good of the whole, from the mere pretender who is working wholly for the dimes of his victims? Who of us that have read the reports of the New York, Brooklyn, Cincinnati, and Philadelphia Societies, that meet once a month or oftener, and have not wished we enjoyed the same privilege? And, when we contrast theirs with our humble efforts, should it discourage us? Rather let it stimulate every one of us to greater efforts, to bring our standard here, up as near as possible to theirs, and, if possible, a little ahead. When we see what has been accomplished by a few earnest men in our local Societies, in the formation of the American Dental Association, and the advanced position that Association has taken, have we not a right to feel grateful that we have (in an humble way) contributed to it? And, as that Association depends upon local Societies for its continuance, should it not encourage us to renewed efforts, to not only keep up our present organization, but to advocate and help to form others? Those of us who were present at the meeting in Cleveland, in 1862, and at the one last July, in Philadelphia, know how much we have to bless the true and earnest men connected with it, and how much the community will be benefitted by the light and truths there set forth. The essays and papers read and contributed by Dr. Atkinson on Dental Pathology and Surgery, Dental Physiology, and Institutes of Dental Science, are worth reading by all of us; and the most of us would do well to study them carefully. Professional Education, a paper read by Dr. J. Foster Flagg, is a valuable production and should be read and studied by every young man who intends to make Dentistry his profession; and a very large portion of us can profit by its study. Dr. W. H. Allen gave us a valuable paper on Irregularities of the Teeth. Exposed Pulps and Alveolar Abscess, was the title of a paper by Dr.

A. C. Hawes, and read by Dr. J. F. Flagg; with which was printed a Resolution, which we think is just suited to the times. The report on Local Societies, by Dr. J. Taft, was just what those of us acquainted with him would expect—precise, and right to the point. Extraction of Teeth, by G. W. Ellis, is a paper that must be read in order to be appreciated; and none of us will read it and say we have not been benefitted; and I would advise all those who are advertising "Teeth extracted without pain" to study it well.

The report on Dental Education, also by Dr. G. W. Ellis, should be on the table of every dentist, and handed to every applicant for dental instruction, with a request that he read it before he makes up his mind to be a dentist.

Dr. J. H. McQuillen's report on Dental Literature, is just what is needed; and his suggestions, if followed out, no doubt, would vastly improve our literature. Although it might keep many of us from appearing in print, yet there is no doubt it would be far better for the whole in the end.

Dr. C. Palmer's Address, together with the instruments he presented to the Association, are not among the least of the benefits we derived by attending its session. By him we can learn how he who commenced over twenty years since, as it were in the back woods, by earnest and persevering effort has come to be one of the best operators; and presented to the Association duplicates of instruments of his own make, the best adapted for the use they were designed for, of any I have yet seen.

Then, the discussions upon the papers read, reported by Dr. G. W. Ellis, though not so full as I could wish, are certainly worth our perusal and study.

And I would here say, that the whole is copyrighted and placed in the hands of a publishing committee, and is now printed, forming a book of 145 pages, which contains more valuable matter for the dental practitioner and student than any other book of its size. And that, with the proceedings of the Association from its first organization, can be had for three dollars; and my advice to all here who have not got it is, without delay, to forward the amount to Dr. J. Taft at Cincinnati, and obtain it.

We also have the American Dental Convention, which meets once a year,—last August, meeting at Saratoga, and was well attended. The papers and essays read were good, and have mostly been published in our dental journals. The discussions were generally of an instructive character; and all appeared to be seeking truths, that they might the better perform their duty to their fellow-beings. And through the efforts of your humble servant, assisted by many friends, the Convention was appointed to be held at Detroit next August; when I hope every one of this Association, and all the rest of the true dentists in the State, and as many from other States as possible, will be present; and show to the friends from the East who have nerve enough to come so far west, that what they call the West, instead of being all a wilderness, is the heart and center of the United States.

And now, when you have read the Transactions of the American Dental Association, and learned all you can of the doings of the American Dental Convention, and then contrast the brighter lights in both with the genius that fashioned the ones from which those I present to you were copied and improved, you will certainly agree with me, when I say, that no profession has advanced so rapidly as ours. And it becomes us not to be idle, or we shall be left far behind in the race we are running with our brother dentists.

I now only have to thank you for the courtesy you all have shown me while I have occupied that Chair, and in listening to my Retiring Address.

